# Safety and Immunogenicity of SII’s 10-Valent Pneumococcal Conjugate Vaccine (PCV10-SII) in Vietnamese Children Aged from 6 Weeks to 24 Months: An Open-Label, Single-Arm Bridging Study

**DOI:** 10.3390/vaccines14040336

**Published:** 2026-04-10

**Authors:** Vu Tung Son, Bui Dang The Anh, Vu Ngoc Hoan, Hoang Van Than, Bui Kim Linh, La Thi Huong Giang, Nguyen Tien Manh, Luong Thi Thu Thao, Hoang Xuan Cuong, Dao Truong Giang, Do Tuan Dat, Le Thi Huong Giang, Sandeep C. Mulay, Vistasp Sethna, Pham Van Hung

**Affiliations:** 1 Department of Military Epidemiology, Vietnam Military Medical University (VMMU), Hanoi 100000, Vietnam; 2 Southern Branch, Vietnam Military Medical University (VMMU), Ho Chi Minh City 700000, Vietnam; 3 Gastroenterology and Hepatology Department, 103 Military Hospital, Vietnam Military Medical University (VMMU), Hanoi 100000, Vietnam; 4 Company for Vaccine and Biological Production No.1 (VABIOTECH), Ministry of Health, Hanoi 100000, Vietnam; 5 Serum Institute of India Pvt. Ltd., 212/2, Off Soli Poonawalla Road Hadapsar, Pune 411028, India

**Keywords:** PCV10-SII pneumococcal conjugate vaccines, Vietnam, vaccine safety, immunogenicity

## Abstract

Background: Pneumococcal conjugate vaccines (PCVs) prevent severe disease in children, but high costs limit access. PCV10-SII (PNEUMOSIL), a 10-valent PCV prequalified by the World Health Organization (WHO) in 2019, offers a cost-effective alternative. This study assessed its safety and immunogenicity in Vietnamese children aged 6 weeks–24 months. Methods: An open-label, single-arm study enrolled 304 children in three age groups: 6 weeks–6 months (n = 151), >6–12 months (n = 76), and >12–24 months (n = 77). Participants received two or three doses. Safety was evaluated through immediate reactions, adverse events (AEs), serious adverse events (SAEs), and withdrawals. Immunogenicity was measured 28 days after the final dose using serotype-specific IgG geometric mean concentrations (GMCs), opsonophagocytic activity (OPA) titers, and seroresponse rates. The trial was approved by the IRB of the National Ethics Council (code: No. 75/CN-HĐĐĐ on date 4 June 2021) and was registered with ClinicalTrials.gov, NCT05140720. Results: Of 304 enrolled participants, 294 (96.7%) completed follow-up. No immediate adverse events or serious adverse events occurred. Unsolicited adverse events were reported in 17%, mainly respiratory, while serious adverse events occurred in 4%. Mild local/systemic reactions (e.g., injection site pain, crying) resolved without sequelae. Immunogenicity was strong, with GMCs 1.8–9.11 µg/mL, GMTs 277.8–22,342, and seroresponse rates >90% for 9 of 10 serotypes, serotype 6B demonstrated a slightly lower seroresponse rate of 88.6%. Conclusions: PCV10-SII demonstrated favorable safety and robust immunogenicity, supporting its inclusion in national immunization programs as an affordable option for pneumococcal disease prevention.

## 1. Introduction

*Streptococcus pneumoniae* (pneumococcus) is a major cause of disease worldwide, ranging from conditions such as otitis media, sinusitis, and bronchitis—which may vary from mild to severe—to life-threatening illnesses including pneumonia, bacteremia, and meningitis, particularly in children under one year of age [1]. *S. pneumoniae* is one of the leading causes of morbidity and mortality globally, being particularly dangerous for children in low- and middle-income countries (LMICs) [2,3,4]. To date, vaccination remains the most effective strategy for reducing both morbidity and mortality caused by pneumococcus [5].

The first Pneumococcal Vaccines, which included antibodies to 14 capsular antigenic serotypes, was approved for use in 1977, followed by the 23-valent vaccine (PPSV23) in 1983. PPSV23 remains widely used in adults, but its poor immunogenicity in infants under 2 years of age necessitated the development of pneumococcal conjugate vaccines (PCVs). The first conjugate formulation, PCV7, was introduced to address this gap, offering protection against seven serotypes responsible for the majority of invasive pneumococcal disease (IPD) in children [6].

Several PCVs are currently available globally. The 10-valent (PCV10-GSK, Synflorix^®^; GSK, Rixensart, Belgium) and 13-valent (PCV13, Prevenar 13^®^; Pfizer, New York, NY, USA) vaccines have been the most widely used, particularly in national immunization programs of LMICs supported by Gavi. More recently, higher-valent formulations have been approved, including PCV15 (Vaxneuvance^®^; Merck & Co., Rahway, NJ, USA), PCV20 (Prevnar 20^®^; Pfizer, New York, NY, USA), and PCV21 (Capvaxive^®^), which offer progressively broader serotype coverage. However, these newer vaccines were developed primarily for use in high-income settings and adult populations—PCV21, for instance, targets serotypes predominant in adults and excludes several common pediatric serotypes such as 6B and 23F—and their substantially higher cost limits their feasibility for routine pediatric immunization programs in LMICs (Table 1) [7,8].

In this context, PCV10-SII (PNEUMOSIL^®^), developed by the Serum Institute of India, represents a cost-effective, WHO-prequalified alternative specifically designed for pediatric use in LMICs. Notably, PCV10-SII includes serotypes 6A and 19A in place of serotypes 4 and 18C compared to PCV10-GSK, making its serotype composition comparable to PCV13 while remaining affordable (Table 1) [9,10].

The clinical development of PCV10-SII included a phase I/II trial evaluating its safety and immunogenicity in adults, toddlers, and infants in The Gambia, which demonstrated an acceptable safety profile across all age groups [11]. However, as PCV10-SII is specifically designed for pediatric use and its serotype composition does not fully reflect the serotype distribution of invasive pneumococcal disease in adults, its use is currently recommended for children only.

In LMICs, PCV10-GSK and PCV13 have been gradually introduced into routine immunization programs with Gavi support; however, uptake remains incomplete in many countries due to supply constraints and cost.

In Vietnam, no domestically produced pneumococcal vaccine exists, and PCVs have not yet been incorporated into the national expanded programme on immunization (EPI). The vaccines currently available in Vietnam include PCV10-GSK, PCV13, PCV20, and PPSV23 (Pneumo 23), all of which are accessed through the private market. Researching and evaluating an affordable PCV10 vaccine is therefore essential to expanding prevention options for individuals at heightened risk, particularly children under 2 years old.

In Vietnam, serotypes 6A and 19A are among the most prevalent causes of invasive pneumococcal disease [9], making PCV10-SII a particularly relevant candidate for the local epidemiological context. To date, no published data exist on the safety or immunogenicity of PCV10-SII in Vietnamese children. Therefore, this study aimed to evaluate the safety and immunogenicity of PCV10-SII administered as a catch-up regimen in vaccine-naive Vietnamese infants and young children aged 6 to 24 months, to support its potential inclusion in national immunization strategies.

## 2. Materials and Methods

### 2.1. Study Design

This was an open-label, single-arm bridging study conducted from June 2021 to June 2023 at Dong Hung ward, Hung Yen province—a rural site located in the North of Vietnam.

The study complied with the Declaration of Helsinki, the protocol, good clinical practices and ethical guidelines for biomedical research on human participants (Viet Nam Council of Medical Research, 2013). The trial was registered with Clinical Trials (NCT05140720). And the trial was approved by Institutional Ethics Committees within Vietnam’s Ministry of Health (code: No. 75/CN-HĐĐĐ on date 4 June 2021). Each participant’s legal representative provided written informed consent before study participation.

### 2.2. Participants

Participants were stratified into three age groups (6 weeks–6 months, >6–12 months, and >12–24 months) following the WHO-recommended catch-up schedules. Older children required fewer doses due to a more mature immune system [12]. Eligible subjects included Vietnamese healthy children aged of either gender from 6 weeks to 24 months. Exclusion criteria comprised prior receipt of any pneumococcal vaccine, culture-confirmed pneumococcal disease, immunodeficiency, or a documented history of hypersensitivity to any investigational vaccine component. Participants were also excluded if they had received another vaccine, serum, or blood product within the preceding 30 days; presented with a body temperature ≥37.0 °C or any acute infection; had received immunomodulating agents within the past six months; or were undergoing concomitant treatment with antimalarial drugs. For administration of the second and third doses, exclusion criteria additionally included the occurrence of any serious adverse event related to the study vaccination protocol or the emergence of any new condition meeting the initial exclusion criteria.

### 2.3. Vaccination Process

In this study, a 0.5 mL dose of the SIIPL-PCV (PCV10-SII) vaccine, lot numbers 2082Y001A and 2092Y001C, was administered. The vaccination regimen followed the dosing schedule approved by the World Health Organization (WHO) as part of the prequalification process. Prior to implementation, the dose and regimen were reviewed and approved by the Ministry of Health of Vietnam. All vaccinations were conducted under the supervision of trained healthcare professionals in a controlled clinical setting.

### 2.4. Clinical Trial Objectives and Endpoints

The primary objective of the study was to characterize the safety profile of PCV10-SII in enrolled participants. Safety endpoints included: (i) the proportion and severity of immediate adverse events occurring within 30 min after vaccine administration; (ii) the proportion and severity of solicited local and systemic adverse reactions during the first 7 days post-vaccination; and (iii) the proportion and severity of unsolicited adverse events and serious adverse events (SAEs) throughout the study period. Participant compliance with study procedures was also monitored.

Immunogenicity was assessed by pneumococcal serotype-specific immune response 28 days after the last dose of PCV10-SII. The first endpoints were the proportion of participants with seroresponse (serotype-specific IgG concentrations ≥ 0.35 µg/mL or OPA titer ≥ 1:8). The second endpoints were geometric mean concentrations and titres (GMC and GMT) measured 28 days after the last vaccination.

### 2.5. Follow-Up and Data Collection

Participants meeting the inclusion criteria and without any exclusion criteria were enrolled and randomized by investigators to receive PCV10-SII. Following enrolment, baseline demographic characteristics—including date of birth, sex, weight, and height—were collected using a standardized questionnaire completed by the study physician. Prior to vaccination, each child’s medical history was reviewed and a physical examination was performed to exclude contraindications to immunization.

Participants were stratified into three age groups. Infants aged 6 weeks to 6 months received three doses of PCV10-SII. Children aged >6 to 12 months received two doses administered at a 1-month interval. Children aged >12 to 24 months received two doses administered at a 2-month interval.

Blood samples were collected prior to vaccination and 28 days after the final dose. Samples were stored and transported at 2–8 °C. Serum concentrations of anticapsular immunoglobulin G (IgG) against the 10 pneumococcal serotypes included in PCV10-SII were quantified using enzyme-linked immunosorbent assay (ELISA) at the Murdoch Children’s Research Institute, Australia. Functional immune responses were assessed using a four-fold multiplexed opsonophagocytic assay (OPA) [13,14]. Seroresponse was defined as an IgG concentration ≥0.35 µg/mL, a threshold associated with protection against invasive pneumococcal disease (IPD) in infants; functional seroresponse was defined as a reciprocal OPA titer ≥8 [15,16]. Clinical data management and statistical analyses were performed by VietStar Biomedical Research, Hanoi, Vietnam.

### 2.6. Safety Assessment

All participants were monitored for safety from the administration of the first dose until 28 days after the final vaccination. Adverse events (AEs) were documented throughout this period. Immediately following each vaccination, participants were observed for 30 min to identify any acute reactions. Participants aged >6 months to ≤24 months attended three scheduled clinic visits, whereas those aged 6 weeks to 6 months attended four visits. A visit window of ±2 weeks was permitted. At each visit, participants underwent a physical examination, and parents or guardians were interviewed regarding adverse events and concomitant medication. Parents or guardians were instructed to report any serious adverse event (SAE) to the investigator without delay.

Injection-site and systemic reactogenicity was solicited in clinic at 30 min post-vaccination and daily for 7 days thereafter, with severity graded on a four-point scale according to protocol definitions. Any reactogenicity persisting beyond the follow-up period was documented as an unsolicited adverse event (AE) and monitored accordingly. Unsolicited AEs were recorded from the time of consent until 28 days after vaccination. All adverse events were categorized using MedDRA version 25.1 (International Council for Harmonisation of Technical Requirements for Pharmaceuticals for Human Use, Geneva, Switzerland) and graded for severity (mild, moderate, severe, or life-threatening) based on pre-specified definitions. All solicited local and systemic adverse events were presumed to be vaccine-related. Causality assessment of unsolicited AEs was performed by investigators using clinical judgment, with classification as very likely/certain, probable, possible, unlikely, unrelated, or unclassifiable.

Health staff received training in the documentation of solicited adverse events, unsolicited adverse events, and serious adverse events (SAEs). Measuring scales were provided within a structured diary to facilitate standardized recording. Detailed safety information for each AE was collected through subject diary cards and supplemented by telephone contact with study staff. All safety data were reviewed at each scheduled visit.

Serious adverse events (SAEs) were reported to the Institutional Ethics Committees and reviewed by an independent Data and Safety Monitoring Board within the stipulated timelines. All medical expenses and hospital visits related to SAEs were covered by the study sponsor.

### 2.7. Immunogenicity Assessment

The evaluable immunogenicity population comprised eligible participants who received all assigned vaccinations, provided blood samples within the required time frames, had at least one valid and determinate assay result for the planned analyses, did not receive prohibited vaccines, and had no major protocol violations. Pneumococcal serotype-specific IgG geometric mean concentrations (GMCs) were calculated for each age group, together with associated 95% confidence intervals (CIs). For each serotype, exact unconditional two-sided 95% CIs were determined. The proportions of participants achieving prespecified IgG concentrations 28 days after the final dose were also evaluated with 95% CIs; these thresholds were defined according to serotype-specific values previously established by ELISA. Opsonophagocytic assay (OPA) geometric mean titers (GMTs) were summarized in a manner analogous to IgG GMCs.

### 2.8. Statistical Analysis

Descriptive statistics were applied to summarize demographic characteristics, seroconversion rates, and adverse events. Geometric mean concentrations (GMCs) and geometric mean titers (GMTs) were reported together with standard deviations (SDs) and 95% CIs. Statistical significance of changes in GMCs and GMTs from baseline was assessed using paired t-tests, with *p*-values < 0.05 considered significant. All analyses were performed using SAS^®^ software, version 9.4 (SAS Institute Inc., Cary, NC, USA).

## 3. Results

### 3.1. Study Participants

**Figure 1:**
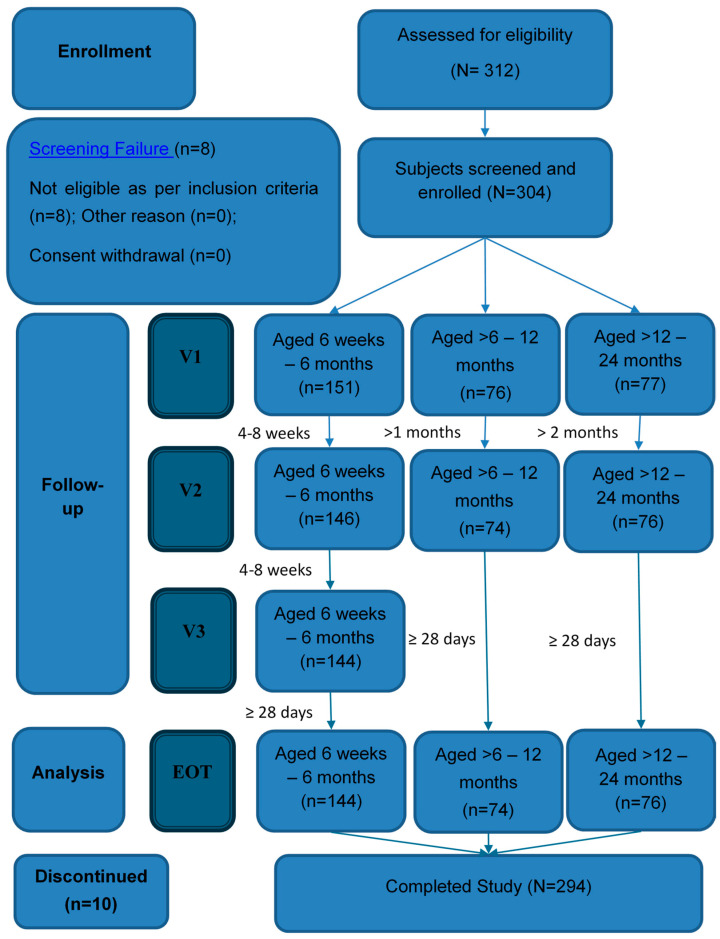
Enrollment flow chart.

Overall, 312 participants were screened, of whom 304 were eligible and enrolled; all 304 (100%) received at least one dose of vaccine. The detailed participant selection and flow throughout the study period are illustrated in Figure 1. Across age groups, completion of all planned doses was achieved by 95.3% of participants aged 6 weeks to 6 months (three-dose schedule) and 98.0% of participants aged >6 to 24 months (two-dose schedule). In total, 294 participants (96.7%) were included in the follow-up analysis conducted 28 days after the final vaccination. The common reasons for exclusion are presented in Figure 1. Baseline demographic characteristics were comparable across age groups (Table 2), with additional demographic breakdowns also provided in Table 2.

No significant differences in demographic characteristics were observed among the three study groups. Increases in height and weight were consistent with expected developmental progress. By study completion, 10 of 304 participants (3.3%) had discontinued participation, primarily due to personal circumstances or loss to follow-up. Notably, no participant withdrew because of safety concerns related to the investigational product. At enrollment, all participants underwent a clinical examination by the investigator, and no significant abnormal findings were detected.

### 3.2. Safety

*Adverse events immediately during 30 min after taking the vaccine:*

No expected or unexpected adverse events, including serious adverse events (SAEs), were observed within 30 min of vaccine administration at any study visit.

*Adverse events during the 7-day period after each of the three-dose visits:*

**Figure 2:**
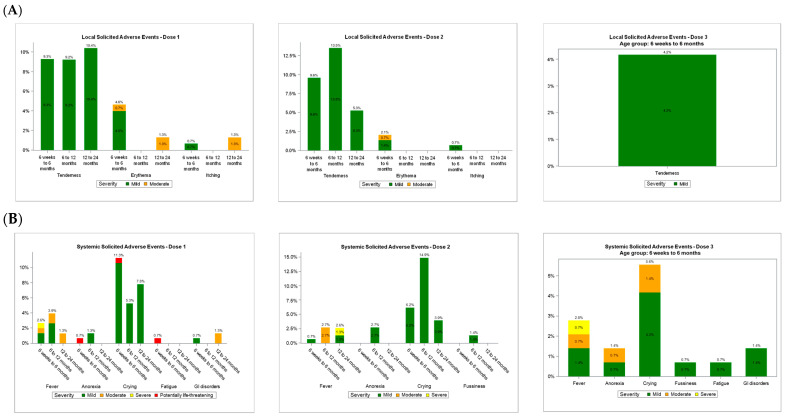
Percentages of participants with reported (**A**) local reactions and (**B**) systemic events following each vaccine dose.

For dose 1, n = 304. For dose 2, n = 296. For dose 3, n = 144. The n values represent the number of participants with any diary data reported after the specified dose (Figure 2). Severity of adverse events was graded by parents or legal guardians according to instructions provided by the investigator staff: mild (Grade 1), moderate (Grade 2), severe (Grade 3), and potentially life-threatening (Grade 4). For redness and swelling, grading was based on the description of the affected area. For pain and all systemic events, grading was determined by the extent to which the event interfered with daily activity. Fever severity was classified by temperature range: mild (38.0–38.4 °C), moderate (38.5–38.9 °C), severe (>39–40 °C), and potentially life-threatening (>40 °C)

Overall, the frequency and severity of local reactions demonstrated a slight decrease with subsequent doses (Figure 2A). Local reactions persisted for a median duration of 1.0–2.0 days after each dose and were predominantly mild or moderate in intensity. Severe local reactions were uncommon, occurring in ≤1.3% of participants (n = 0–1 per age group) following each dose (Table 3). The most frequently reported local adverse event within 7 days of vaccination was tenderness, which occurred at a low rate (<10%) (Table 3, Figure 2A).

**Table 3:** Adverse events assessed to be probably possibly or remotely related to investigational product administration during the 7-day period following each of the three scheduled doses, stratified by age group.

Adverse Event	6 Weeks–6 Months Group (n = 151)			>6–12 Months Group (n = 76)		>12–24 Months Group (n = 77)	
	After the First Time Visit (n = 151)	After the Second Time Visit (n = 146)	After the Third Time Visit (n = 144)	After the First Time Visit (n = 76)	After the Second Time Visit (n = 74)	After the Third Time Visit (n = 77)	After the Second Time Visit (n = 76)
***Local ADEs***							
Tenderness	14 (9.27)	14 (9.59)	6 (4.17)	7 (9.21)	10 (13.51)	8 (10.39)	4 (5.26)
Erythema	7 (4.64)	3 (2.05)	0 (0.0)	0 (0.0)	0 (0.0)	1 (1.29)	0 (0.0)
Itching	1 (0.66)	1 (0.68)	0 (0.0)	0 (0.0)	0 (0.0)	1 (1.29)	(0.0)
***Systemic ADEs***							
Fever	4 (2.64)	1 (0.68)	4 (2.78)	3 (3.95)	2 (2.7)	1 (1.29)	2 (2.64)
Anorexia	1 (0.66)	0 (0.0)	2 (1.39)	1 (1.32)	2 (2.7)	0 (0.0)	0 (0.0)
Crying	17 (11.26)	9 (6.16)	8 (5.6)	4 (5.26)	11 (14.86)	6 (7.79)	3 (3.95)
Fussiness	0 (0.0)	0 (0.0)	1 (0.69)	0 (0.0)	1 (1.35)	0 (0.0)	0 (0.0)
Fatigue	1 (0.66)	0 (0.0)	1 (0.69)	0 (0.0)	0	0 (0.0)	0 (0.0)
GI disorders	1 (0.66)	0 (0.0)	2 (1.39)	0 (0.0)	1 (1.36)	0 (0.0)	0 (0.0)

Similar proportions of participants across age groups reported systemic adverse events (Table 3). The majority of systemic events were mild or moderate in severity, while severe systemic events occurred in ≤1.3% of participants after each dose (Figure 2B). The most frequently reported systemic adverse event within 7 days of vaccination was crying, which was observed at a low rate (<10%) (Table 3, Figure 2B). Fever >39 °C was reported in ≤0.7% of participants after each dose, and no cases of fever >40 °C were observed (Table 3). Individual systemic events persisted for a median duration of 1.0–2.0 days.

A total of 75 unsolicited AEs were reported among the 52 participants. AEs were reported for 31 participants in the 6 weeks to 6 months age groups, 11 participants in the >6 to 12 months, and 10 participants in the >12 to 24 months. Most of AEs belonged to infection and infestations. The majority of AEs were mild (n = 26) or moderate (n = 24) in severity; and all were resolved without any sequelae. There were only 2 unsolicited AEs (gastrointestinal disorders) considered unlikely to be related to PNEUMOSIL.

SAEs were reported in 12 participants. These included infection and infestations; bronchitis; gastrointestinal infection; influenza, and pneumonia. Most of SAEs were of moderate and severe intensity. The majority were unrelated to study vaccines. One SAE (influenza) was considered unlikely related to PNEUMOSIL. No death occurred during the study.

### 3.3. Immunogenicity

Serotype-Specific IgG Concentrations

It can be seen that 28 days after the final dose of study vaccine, at least 90% of participants across all age group achieved a detectable IgG concentrations ≥ 0.35 µg/mL for all vaccine pneumococcal serotypes, with the exception of serotype 6A (85.71%) and serotype 9V (88.57%) in the >6–12 months age group, and serotype 6B, which showed 80% seroprotection in the 6 weeks–6 months group and 85.71% in the >6–12 months age group (respectively). However, the seroresponse rate in the general population exceeded 90% of subjects achieving an IgG concentration ≥ 0.35 µg/mL for all serotypes, except for serotype 6B, which had a slightly lower rate of 88.57% (Table 4).

At 28 days after the last vaccination, over 97% of participants in each serotypes across all age groups achieved seroresponse with evaluation of OPA titers (reciprocal OPA titer ≥ 8), except serotype 6B in the 6 weeks–6 months age group (94.29%) (Table 5).

For all 10 serotypes, IgG GMCs measured 28 days after the last vaccination were generally similar across age groups and exceeded 1 µg/mL, with the highest levels observed for serotype 14. GMCs range from 1.41 to 8.67 µg/mL for participants in the 6 weeks–6 months age group, from 1.3 to 7.97 µg/mL for participants in the >6–12 months age group and from 2.52 to 12.8 µg/mL for participants in the >12–24 months age group (Table 6).

Opsonophagocytic assay (OPA) titers were determined in a subset of participants for each pneumococcal serotype to confirm the functional activity of PCV10-elicited immune responses. At 28 days after the final vaccination, OPA geometric mean titers (GMTs) for the 10 serotypes were largely comparable between participants aged 6 weeks to 6 months and those aged >6 to 12 months (Table 5). In contrast, GMTs across all 10 serotypes were higher in the >12 to 24 months age group following the last dose (Table 7).

## 4. Discussion

This study provides safety and immunogenicity data on SIIPL-PCV, a 10-valent pneumococcal conjugate vaccine formulated with serotypes selected to maximize coverage against pneumococcal disease in Vietnam. The findings demonstrate that PCV10-SII is well tolerated and immunogenic across all three catch-up age groups, with immune responses comparable to those reported in pivotal trials conducted in other geographical regions. In addition, the findings offer preliminary evidence to support the co-administration of SIIPL-PCV within the vaccine registration process in Vietnam.

### 4.1. Safety

PCV10-SII was well tolerated in all study participants. Local reaction and systemic events were mostly mild or moderate, transient in nature, and did not increase in frequency with subsequent doses. Within age groups, tenderness was the most common solicited local symptom, with crying as the most common solicited systemic symptom; these were mild to moderate in severity, self-limited, and considered related to PCV10-SII. Reports of severe in severity-solicited symptoms were infrequent. AEs occurred at similar frequencies across groups and reflected common medical events or conditions for this age range. The incidence of local and systemic AEs was notably lower compared with findings from other studies of the same vaccine [17]. This favorable safety profile is consistent with data reported from the phase I/II and phase III trials of PCV10-SII conducted in The Gambia, where local and systemic reactions were similarly mild and transient [11,18], as well as with post-licensure surveillance data from the phase III 2+1 schedule trial of the same vaccine [17].

Twelve participants reported SAEs, of which only 1 was considered unlikely to be related to the vaccine (influenza). During this study period, which coincided with seasonal transitions in Vietnam, most hospitalized participants were diagnosed with pneumonia. This trend can be attributed to the vulnerability of children aged 6 to 24 months, who are particularly susceptible to progressing from rhinopharyngitis to pneumonia during this time. The occurrence of pneumonia-related hospitalizations during the study period should be interpreted in the context of Vietnam’s seasonal respiratory disease burden rather than as a vaccine-related signal. There were no study withdrawals; no protocol violations were reported.

A small proportion of study participants experienced unexpected adverse events (AEs); however, the majority of unsolicited AEs were considered unrelated to PCV10-SII. Notably, only two unsolicited AEs—both gastrointestinal disorders—were assessed as unlikely to be related to PCV10-SII, primarily due to their onset occurring 7 days post-vaccination. Although unsolicited AEs and SAEs occurring within seven days post-vaccination were considered temporally associated with the investigational vaccine, this association alone does not necessarily imply a causal relationship. Evaluation of the causal relationship between AEs and the investigational product was conducted according to established principles of causality assessment by trained investigators, ensuring accuracy and objectivity.

### 4.2. Immunogenicity

PCV10 catch-up regimens in Vietnam in vaccine-naive older infants and young children resulted in a robust immune response to all vaccine serotypes.

Robust immune responses to all 10 vaccine serotypes were elicited following the PCV10 regimen, as evidenced by serotype-specific IgG geometric mean concentrations (GMCs) and the proportion of participants achieving prespecified IgG thresholds 28 days after the final vaccination. In the general population, seroresponse rates exceeded 90% for all serotypes, with the exception of serotype 6B, which demonstrated a slightly lower rate of 88.6% and as low as 80% in the youngest age group. This pattern is well documented across multiple PCV trials regardless of vaccine formulation. In both the phase I/II and phase III trials of PCV10-SII conducted in The Gambia, seroresponse rates for serotype 6B were similarly below the 90% threshold [11,18]. Comparable findings have been reported for PCV10-GSK and PCV13 in various populations, where serotype 6B consistently demonstrates lower IgG immunogenicity relative to other serotypes [19,20]. The lower immunogenicity of serotype 6B is thought to reflect its distinct capsular polysaccharide structure and cross-reactivity patterns [21]. Notably, serotype 6B prevalence in Vietnam is lower relative to other serotypes [9], suggesting that the modest seroresponse rate for this serotype is unlikely to substantially affect overall vaccine effectiveness in the local epidemiological context.

To further contextualize these findings, Table 5 and Table 6 present a comparison of IgG GMCs and OPA GMTs observed in the current study against published data from the phase I/II and phase III trials of PCV10-SII in The Gambia [11,18], as well as selected studies of PCV10-GSK and PCV13 conducted in Vietnamese infants [19], providing a directly relevant regional comparator. Overall, observed IgG GMCs and OPA GMTs across all serotypes in the present study were comparable to values reported in these trials, confirming the consistent immunogenic performance of PCV10-SII across diverse study populations and supporting its generalizability to the Vietnamese pediatric context.

Functional immune responses, as measured by opsonophagocytic assay (OPA), demonstrated patterns consistent with IgG responses. Moreover, the proportion of participants with functional OPA antibodies (titers ≥8) exceeded 94%, including for serotype 6B. This is a particularly important finding, as OPA titers more directly reflect functional bactericidal activity and correlate more closely with clinical protection than IgG concentrations alone [13,15,16]. The high OPA response rate for serotype 6B—despite its lower IgG seroresponse—suggests that functional protection is effectively maintained, and that IgG GMC alone may underestimate the true protective response for this serotype. These OPA findings were comparable to those reported in the phase I/II and phase III trials of PCV10-SII in The Gambia [11,18].

Antibody concentrations were generally higher among the oldest children at 28 days after the final vaccination. While IgG GMCs were higher for eight serotypes in the 12–24 month age group, IgG GMCs for serotypes 1 and 7F remained lower compared with younger age groups. The reason for this age-specific pattern for serotypes 1 and 7F is not fully understood; however, it may reflect differential prior antigen exposure or immunological priming in younger infants [20]. The more robust immunologic responses observed in older children are consistent with the natural maturation of the immune system, which becomes more efficient with age, leading to stronger vaccine-induced responses. Overall, post-vaccination IgG antibody levels were generally higher in the older age group, which may be suggestive of prior exposure to *S. pneumoniae* [22].

Importantly, older children who received a 2-dose PCV10 regimen achieved immune responses comparable to those observed in younger infants receiving a 3-dose regimen. These findings support the appropriateness of the WHO-recommended age-stratified catch-up dosing schedule and provide evidence that a reduced number of doses can elicit adequate protection in older vaccine-naive children [12].

### 4.3. Limitations

A key limitation of this study is the short-term follow-up, with immunogenicity assessed only 28 days after the final dose. Long-term persistence of immunity was not evaluated and warrants further investigation. The absence of a booster dose assessment is also a limitation, as waning immunity over time cannot be excluded. Additionally, the current study did not include a control group, which precludes direct head-to-head comparison with other licensed PCV formulations. Data on pre-term infants and children with underlying medical conditions were also not captured. These gaps should be addressed in future post-licensure surveillance and real-world effectiveness studies.

## 5. Conclusions

This study provides sufficient evidence supporting the safety and immunogenicity of PCV10-SII in Vietnamese children. The data generated herein form a critical basis for evaluation by the national vaccine regulatory authority and will support approval and marketing authorization of the vaccine in Vietnam.

## Figures and Tables

**Table 1:** Comparison of serotype composition between PCV10-SII and other licensed pneumococcal conjugate vaccines.

Vaccine	Number of Serotypes	Serotypes Included
PCV10-GSK (Synflorix)	10	1, 4, 5, 6B, 7F, 9V, 14, 18C, 19F, 23F
PCV10-SII (Study Vaccine)	10	1, 5, 6B, 7F, 9V, 14, 19F, 23F, 6A, 19A
PCV13 (Prevenar 13)	13	1, 3, 4, 5, 6A, 6B, 7F, 9V, 14, 18C, 19A, 19F, 23F
PCV15 (Vaxneuvance)	15	PCV13 + 22F, 33F
PCV20 (Prevnar 20)	20	PCV15 + 8, 10A, 11A, 12F, 15B
PCV21 (Capvaxive)	21	3, 6A, 7F, 8, 9N, 10A, 11A, 12F, 15A, 15C, 16F, 17F, 19A, 20A, 22F, 23A, 23B, 24F, 31, 33F, 35B

**Table 2:** Demographic characteristics of Enrolled Participants (n = 304).

Characteristics	Group 6 Weeks to 6 Months (n = 151)	Group >6 to 12 Months (n = 76)	Group >12 to 24 Months (n = 77)
Age (days) (n = 304)			
Mean (SD)	98.01 (42.92)	291.39 (42.68)	541.75 (102.00)
Median	99.00	293.00	537.00
Min–Max	44.00–179.00	193.00–359.00	371.00–758.00
Gender (%)			
Male	84 (55.63%)	32 (42.11%)	38 (49.35%)
Female	67 (44.37%)	44 (57.89%)	39 (50.65%)
Weight, kg (n = 304)			
Mean (SD)	6.29 (1.09)	8.49 (0.97)	10.28 (1.46)
Height, cm (n = 304)			
Mean (SD)	60.83 (4.08)	70.92 (2.84)	79.54 (4.35)
Discontinued (10/304)	3.3%		

**Table 4:** Sero-response rate within IgG by ELISA 28 days after last vaccination.

Serotype	Aged Group			Total (n = 105)
	6 Weeks–6 Months (n = 35)	>6–12 Months (n = 35)	>12–24 Moths (n = 35)	
	n (%) [95% CI]	n (%) [95% CI]	n (%) [95% CI]	n (%) [95% CI]
1	35 (100.0%)	35 (100.0%)	35 (100.0%)	105 (100.0%)
5	35 (100.0%)	35 (100.0%)	35 (100.0%)	105 (100.0%)
6A	33 (94.29%) [78.65–99.30%]	30 (85.71%) [68.77–95.19%]	35 (100.0%)	98 (93.33%) [86.37–97.28%]
6B	28 (80.00%) [62.39–91.56%]	30 (85.71%) [68.77–95.19%]	35 (100.0%)	93 (88.57%) [80.64–93.95%]
7F	35 (100.0%)	35 (100.0%)	35 (100.0%)	105 (100.0%)
9V	32 (91.43%) [75.38–98.20%]	31 (88.57%) [72.06–96.80%]	34 (97.14%) [81.35–99.93%]	97 (92.38%) [85.20–96.65%]
14	35 (100.0%)	35 (100.0%)	35 (100.0%)	105 (100.0%)
19A	33 (94.29%) [78.65–99.30%]	35 (100.0%)	35 (100.0%)	103 (98.10%) [92.34–99.77%]
19F	35 (100.0%)	35 (100.0%)	35 (100.0%)	105 (100.0%)
23F	33 (94.29%) [78.65–99.30%]	34 (97.14%) [81.35–99.93%]	35 (100.0%)	102 (97.14%) [91.17–99.41%]

**Table 5:** Response rate within OPA titers 28 days after last vaccination.

Serotype	Aged Group			Total (n = 105)
	6 Weeks–6 Months (n = 35)	>6–12 Months (n = 35)	>12–24 Moths (n = 35)	
	n (%) [95% CI]	n (%) [95% CI]	n (%) [95% CI]	n (%) [95% CI]
1	34 (97.14%) [81.35–99.93%]	35 (100.0%)	34 (97.14%) [81.35–99.93%]	103 (98.10%) [92.34–99.77%]
5	35 (100.0%)	35 (100.0%)	35 (100.0%)	105 (100.0%)
6A	35 (100.0%)	34 (97.14%) [81.35–99.93%]	35 (100.0%)	104 (99.05%) [93.27–99.98%]
6B	33 (94.29%) [78.65–99.30%]	34 (97.14%) [81.35–99.93%]	34 (97.14%) [81.35–99.93%]	101 (96.19%) [89.96–98.95%]
7F	35 (100.0%)	35 (100.0%)	35 (100.0%)	105 (100.0%)
9V	35 (100.0%)	34 (97.14%) [81.35–99.93%]	35 (100.0%)	104 (99.05%) [93.27–99.98%]
14	35 (100.0%)	35 (100.0%)	35 (100.0%)	105 (100.0%)
19A	35 (100.0%)	35 (100.0%)	35 (100.0%)	105 (100.0%)
19F	34 (97.14%) [81.35–99.93%]	35 (100.0%)	34 (97.14%) [81.35–99.93%]	103 (98.10%) [92.34–99.77%]
23F	35 (100.0%)	34 (97.14%) [81.35–99.93%]	35 (100.0%)	104 (99.05%) [93.27–99.98%]

**Table 6:** Pneumococcal IgG GMCs (µg/mL) 28 days after last vaccination.

Serotype	Aged Group			Total (n = 103)
	6 Weeks–6 Months (n = 35)	>6–12 Months (n = 35)	>12–24 Moths (n = 35)	
	GMC (95% CI)	GMC (95% CI)	GMC (95% CI)	GMC (95% CI)
1	6.81 (5.14–9.01)	5.28 (4.07–6.86)	4.03 (2.95–5.51)	5.25 (4.45–6.19)
5	2.39 (1.86–3.06)	2.69 (2.16–3.36)	2.52 (2.00–3.18)	2.53 (2.22–2.89)
6A	2.99 (1.97–4.55)	1.58 (1.04–2.38)	5.90 (3.98–8.73)	3.03 (2.36–3.89)
6B	1.41 (0.87–2.29)	1.74 (1.17–2.58)	6.00 (4.15–8.66)	2.45 (1.88–3.19)
7F	4.45 (3.29–6.03)	5.02 (4.14–6.10)	4.6 (3.31–6.40)	4.69 (4.00–5.49)
9V	1.75 (1.23–2.49)	1.3 (0.88–1.91)	2.56 (1.91–3.44)	1.80 (1.47–2.20)
14	8.67 (5.86–12.82)	6.82 (5.03–9.25)	12.8 (9.03–18.14)	9.11 (7.45–11.15)
19A	1.97 (1.42–2.73)	3.29 (2.49–4.36)	6.27 (4.61–8.54)	3.44 (2.83–4.17)
19F	7.58 (5.32–10.80)	7.97 (5.98–10.63)	10.83 (7.14–16.42)	8.68 (7.10–10.62)
23F	2.65 (1.8–3.91)	2.87 (2.00–4.14)	5.52 (4.06–7.51)	3.48 (2.83–4.28)

**Table 7:** Pneumococcal OPA GMTs 28 days after last vaccination.

Serotype	Aged Group			Total (n = 105)
	6 Weeks–6 Months (n = 35)	>6–12 Months (n = 35)	>12–24 Moths (n = 35)	
	GMT (95% CI)	GMT (95% CI)	GMT (95% CI)	GMT (95% CI)
1	209.4 (137.1–319.9)	306.2 (208.9–449.0)	334.2 (201.0–555.6)	277.8 (216.5–356.4)
5	656.5 (470.7–915.8)	716.5 (528.0–972.4)	1119 (830.9–1507)	807.4 (674.4–966.7)
6A	3219 (2277–4551)	2707 (1526–4802)	8527 (6177–11,771)	4204 (3245–5446)
6B	2272 (1112–4641)	2267 (1299–3953)	4913 (2723–8864)	2935 (2058–4188)
7F	22,979 (17,089–30,901)	12,618 (9161–17,381)	38,463 (27,822–53,174)	22,342 (18,363–27,184)
9V	1077 (778.6–1489)	1935 (1094–3424)	5503 (4202–7207)	2255 (1733–2935)
14	9485 (6321–14,231)	7706 (5230–11,356)	21,745 (16,134–29,307)	11,670 (9342–14,579)
19A	587.3 (435.1–792.6)	1229 (858.7–1759)	1535 (1164–2025)	1035 (853.7–1254)
19F	5277 (2959–9408)	2645 (1719–4068)	6898 (3536–13,456)	4583 (3307–6351)
23F	8825 (6548–11,894)	4210 (2293–7728)	16,247 (11,878–22,222)	8451 (6496–10,995)

## Data Availability

Data is available at corresponding authors upon reasonable request.

## Abbreviations

The following abbreviations are used in this manuscript:

PCVs	Pneumococcal conjugate vaccines
WHO	World Health Organization
AEs	adverse events
SAEs	serious AEs
GMCs	geometric mean concentrations
OPA	opsonophagocytic activity
SII	Serum Institute of India Pvt. Ltd.
GMT	geometric mean titres
IgG	immunoglobulin G
ELISA	enzyme-linked immunosorbent assay
IPD	invasive pneumococcal disease
SDs	standard deviations
CIs	confidence intervals

## References

[1] Centers for Disease Control and Prevention (2021). Active Bacterial Core Surveillance Report, Emerging Infections Program Network, Streptococcus pneumoniae.

[2] Troeger C., Blacker B., Khalil I.A., Rao P.C., Cao J., Zimsen S.R.M., Albertson S.B., Deshpande A., Farag T., Abebe Z. (2018). Estimates of the global, regional, and national morbidity, mortality, and aetiologies of lower respiratory infections in 195 countries, 1990–2016: A systematic analysis for the Global Burden of Disease Study 2016. Lancet Infect. Dis..

[3] World Health Organization (2005). Bulletin of the World Health Organization.

[4] Black R.E., Cousens S., Johnson H.L., Lawn J.E., Rudan I., Bassani D.G., Jha P., Campbell H., Walker C.F., Cibulskis R. (2010). Global, regional, and national causes of child mortality in 2008: A systematic analysis. Lancet.

[5] Wahl B., O’Brien K.L., Greenbaum A., Majumder A., Liu L., Chu Y., Lukšić I., Nair H., McAllister D.A., Campbell H. (2018). Burden of *Streptococcus pneumoniae* and Haemophilus influenzae type b disease in children in the era of conjugate vaccines: Global, regional, and national estimates for 2000–15. Lancet Globle Health.

[6] Committee on Infectious Diseases (2000). Policy statement: Recommendations for the prevention of pneumococcal infections, including the use of pneumococcal conjugate vaccine (Prevnar), pneumococcal polysaccharide vaccine, and antibiotic prophylaxis. Pediatrics.

[7] Kobayashi M., Farrar J.L., Gierke R., Britton A., Childs L., Leidner A.J., Campos-Outcalt D., Morgan R.L., Long S.S., Talbot H.K. (2022). Use of 15-Valent Pneumococcal Conjugate Vaccine and 20-Valent Pneumococcal Conjugate Vaccine Among U.S. Adults: Updated Recommendations of the Advisory Committee on Immunization Practices—United States, 2022. Morb. Mortal. Wkly. Rep..

[8] Shirley M. (2023). 20-Valent Pneumococcal Conjugate Vaccine: Pediatric First Approval. Pediatr. Drugs.

[9] Tao X., Sharma K., King C., Nguyen T.T., Nguyen T.-A., Dang H.T.T., Duong L.T., Duong T.H.M., Williams P.C., Jayasinghe S. (2026). Prevalence and serotype distribution of nasopharyngeal carriage of *Streptococcus pneumoniae* in Vietnam: A systematic review and meta-analysis. Lancet Reg. Health–West. Pac..

[10] Koshal S.S., Ray A., Hora R., Kaur A., Quadri S.F., Mehra R., Kumari A., Haldar P., Roy A.D. (2023). Critical factors in the successful expansion of Pneumococcal Conjugate Vaccine in India during the COVID-19 pandemic. Vaccine X.

[11] Clarke E., Bashorun A.O., Okoye M., Umesi A., Hydara M.B., Adigweme I., Dhere R., Sethna V., Kampmann B., Goldblatt D. (2020). Safety and immunogenicity of a novel 10-valent pneumococcal conjugate vaccine candidate in adults, toddlers, and infants in The Gambia—Results of a phase 1/2 randomized, double-blinded, controlled trial. Vaccine X.

[12] Organisation World Health Organization (2019). Pneumococcal conjugate vaccines in infants and children under 5 years of age: WHO position paper–February 2019. Wkly. Epidemiol. Rec..

[13] Romero-Steiner S., Frasch C., Concepcion N., Goldblatt D., Käyhty H., Väkeväinen M., Laferriere C., Wauters D., Nahm M.H., Schinsky M.F. (2003). Multilaboratory evaluation of a viability assay for measurement of opsonophagocytic antibodies specific to the capsular polysaccharides of *Streptococcus pneumoniae*. Clin. Vaccine Immunol..

[14] Goldblatt D., Plikaytis B., Akkoyunlu M., Antonello J., Ashton L., Blake M., Burton R., Care R., Durant N., Feavers I. (2011). Establishment of a new human pneumococcal standard reference serum, 007sp. Clin. Vaccine Immunol..

[15] Jódar L., Butler J., Carlone G., Dagan R., Goldblatt D., Käyhty H., Klugman K., Plikaytis B., Siber G., Kohberger R. (2003). Serological criteria for evaluation and licensure of new pneumococcal conjugate vaccine formulations for use in infants. Vaccine.

[16] Rose C.E., Romero-Steiner S., Burton R.L., Carlone G.M., Goldblatt D., Nahm M.H., Ashton L., Haston M., Ekström N., Haikala R. (2011). Multilaboratory comparison of *Streptococcus pneumoniae* opsonophagocytic killing assays and their level of agreement for the determination of functional antibody activity in human reference sera. Clin. Vaccine Immunol..

[17] Adigweme I., Futa A., Saidy-Jah E., Edem B., Akpalu E., Dibbasey T., Sethna V., Dhere R., Kampmann B., Bengt C. (2023). Immunogenicity and safety of a 10-valent pneumococcal conjugate vaccine administered as a 2 + 1 schedule to healthy infants in the Gambia: A single-centre, double-blind, active-controlled, randomised, phase 3 trial. Lancet Infect. Dis..

[18] Clarke E., Bashorun A., Adigweme I., Hydara M.B., Umesi A., Futa A., Ochoge M., Obayemi D., Edem B., Saidy-Jah E. (2021). Immunogenicity and safety of a novel ten-valent pneumococcal conjugate vaccine in healthy infants in The Gambia: A phase 3, randomised, double-blind, non-inferiority trial. Lancet Infect. Dis..

[19] Temple B., Toan N.T., Dai V.T.T., Bright K., Licciardi P.V., Marimla R.A., Nguyen C.D., Uyen D.Y., Balloch A., Huu T.N. (2019). Immunogenicity and reactogenicity of ten-valent versus 13-valent pneumococcal conjugate vaccines among infants in Ho Chi Minh City, Vietnam: A randomised controlled trial. Lancet Infect. Dis..

[20] Trueck J., Snape M.D., Tatangeli F., Voysey M., Yu L.-M., Faust S.N., Heath P.T., Finn A., Pollard A.J. (2014). Pneumococcal serotype-specific antibodies persist through early childhood after infant immunization: Follow-up from a randomized controlled trial. PLoS ONE.

[21] Gening M., Kurbatova E.A., Nifantiev N. (2021). Synthetic analogs of *Streptococcus pneumoniae* capsular polysaccharides and immunogenic activities of glycoconjugates. Russ. J. Bioorg. Chem..

[22] Janapatla R.P., Hsu M.-H., Chen C.-L., Wei S.-H., Yu M.-J., Su L.-H., Lin T.-Y., Chiu C.-H. (2020). Persistence of immunity in children immunised with 13-valent pneumococcal conjugate vaccine and impact on nasopharyngeal carriage: A cross-sectional study. Thorax.

